# Newborns’ attention to socio-emotional prosodic cues from voices

**DOI:** 10.1016/j.isci.2025.113939

**Published:** 2025-11-05

**Authors:** Valentina Silvestri, Silvia Polver, Maria Lorella Giannì, Angelo Petrelli, Matteo Santini, Lorenzo Colombo, Hermann Bulf

**Affiliations:** 1 Department of Psychology, University of Milano-Bicocca, Milan, Italy; 2 Integrative Neuroscience and Cognition Center, UMR8002, Université Paris Cité and CNRS, Paris, France; 3 Laboratoire des Systèmes Perceptifs, UMR CNRS 8248, Ecole Normale Supérieure, PSL University, Paris, France; 4 Department of Developmental and Social Psychology, University of Padua, Padua, Italy; 5 Department of Clinical Sciences and Community Health, University of Milan, Milan, Italy; 6 Fondazione IRCCS Ca' Granda Ospedale Maggiore Policlinico, NICU, Milan, Italy; 7 NeuroMI, Milan Center for Neuroscience, Milan, Italy

**Keywords:** Psychology

## Abstract

Voices are among the most salient stimuli for social interactions. From birth, we can process both linguistic and socio-emotional aspects of voices, but it is not clear how these cues contribute to voice processing. Using non-nutritive sucking, we examined newborns’ attention to a nursery rhyme narrated by an actress, hummed by the same actress, and narrated by a synthesizer, thereby manipulating the weight of linguistic and socio-emotional cues. The role of prenatal auditory experiences in newborns’ response and the relation between sucking variables as well as the cognitive underlying mechanisms were also explored. Results showed an inhibition of sucking behavior in the absence of socio-emotional prosodic cues and a relationship between sucking behavior and the prenatal exposure to maternal speech and music. Moreover, sucking variables clustered into two dimensions of effort and temporal regulation. Overall, these results underscore the role of socio-emotional prosodic cues in early voice processing and its relation with prenatal auditory experience.

## Highlights

• Newborns detect and respond to violations in socio-emotional prosodic cues in voices
• Newborns’ processing of voices is shaped by prenatal experience with speech and music
• Non-nutritive sucking is sensitive to variations in newborns’ attention
• Sucking variables clustered into two dimensions of effort and temporal regulation

## Graphical abstract

**Figure:**
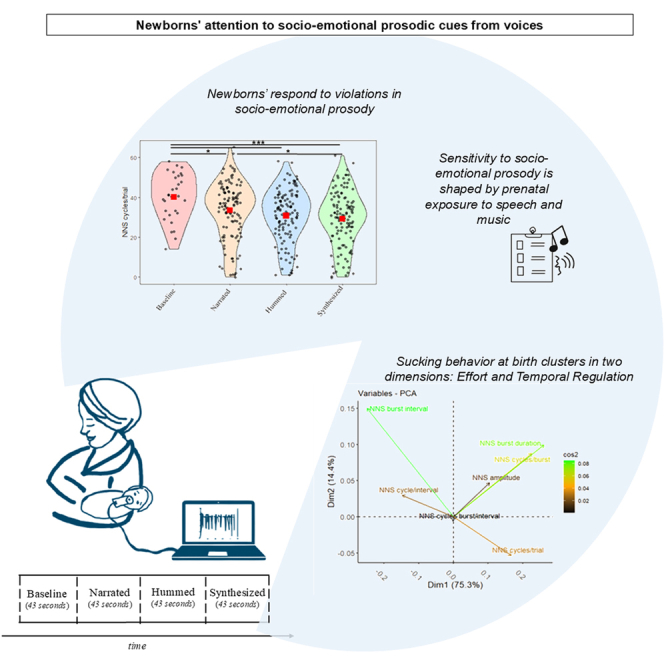

## Introduction

From the moment we are born, awareness of the world around us is surprisingly sharp and many studies have shown that newborns are far more engaged in processing their surroundings than we previously thought.^1^^,^^2^^,^^3^ Among the distal senses, one of the earliest systems to develop is the auditory system, which becomes functional as early as the 24th to 28th week of gestation.^4^^,^^5^ Thus, before birth, fetuses begin to develop the ability to perceive sounds from their surroundings. This prenatal exposure plays a crucial role in shaping their perceptual abilities, laying the groundwork for newborns to adapt to and navigate the world after birth.^6^^,^^7^^,^^8^ For instance, newborns show decreased heart rate in response to short melodies they heard prenatally, with this calming effect lasting for at least six weeks after birth.^9^ Similarly, they prefer a story that the mother had read aloud during pregnancy^10^ and their mother’s voice.^1^^,^^11^ Moreover, newborns can detect rhythms from both music and speech,^12^^,^^13^ prefer speech over non-speech stimuli,^13^ and prefer their native language and rhythmically similar languages over rhythmically dissimilar languages.^14^

Within this context, it is important to recognize that prenatal auditory experiences differ significantly from those after birth. Inside the womb sounds are filtered and attenuated by the properties of maternal tissues and surrounding fluids, which act as low-pass filters.^15^^,^^16^ Consequently, fetuses primarily hear sounds below 400–600 Hz, making it challenging to perceive fine-grained acoustic details, among which many phonemic distinctions. However, prosodic elements such as melody and rhythm are largely preserved, allowing fetuses to process the speech’s overall intonation and rhythm, cues that contribute in organizing the vocal stream into units in a hierarchical way.^17^^,^^18^ It has been proposed that prosodic cues, accessible during gestation, provide a foundational framework for postnatal perception.^19^ In turn, this continuity from prenatal exposure to postnatal perception may explain newborns’ sophisticated abilities to process the prosodic information embedded in linguistic streams.^20^^,^^21^^,^^22^^,^^23^ Notably, prosodic features play a dual role: on one hand, they serve linguistic purposes, such as distinguishing between questions and statements; on the other, they act as a channel for emotional communication through the modulation of features like pitch, intensity, and rhythm.^24^^,^^25^

Evidence of sensitivity to pitch contours and utterance-level prosody at birth supports the notion that prosodic cues are crucial in helping newborns learn words and identify phrases and sentences.^20^^,^^26^ For example, newborns and infants can use prosodic and pitch contours to segment speech,^27^^,^^28^^,^^29^ words,^30^^,^^31^ and to infer the basic word order of their native language.^32^ Moreover, newborns are responsive to affective states conveyed through prosody, such as happiness or sadness.^33^^,^^34^^,^^35^ This attunement to emotional prosody has been reported as early as the 37th week of gestation in the form of a neural mismatch response, an early form of adult mismatch negativity, triggered by positive emotional vocal prosody.^36^ These responses keep refining during the first year of life, reflecting the interplay between innate mechanisms and postnatal experience.^36^^,^^37^ Overall, these findings highlight that prosodic cues mediate both the structure of speech, helping listeners identify linguistic units, and the socio-emotional dynamics of communication, conveying affective information, such as the speaker’s mood or intention. In the present study, the term “socio-emotional prosody” refers to prosodic modulations that convey affective tone and communicative intent.^19^^,^^24^ These cues are embedded in natural speech and constitute some of the earliest signals available for social interaction.^34^ In this sense, the term does not imply the presence of complex social emotions (e.g., guilt or shame), but rather emphasizes how prosody helps regulate early communicative exchanges and infants’ attention.

While the role of linguistic prosodic cues in supporting linguistic processing has been investigated from the first hours of life,^14^^,^^18^^,^^20^ less is known about the role of socio-emotional prosodic information in shaping newborns’ attentional responses to speech. In this study, capitalizing on naturalistic stimuli, we explore the role of socio-emotional vs. linguistic prosodic cues in influencing newborns’attentional mechanisms in response to a nursery rhyme. Specifically, we sought to explore the role of these cues in guiding newborns’ attentional arousal. Attention in newborns can be assessed through the non-nutritive sucking (NNS) measure that takes advantage of the naturally occurring sucking behavior, a simple and rhythmic motor reflex present in all healthy infants.^38^ Specifically, the NNS is a pattern, in the absence of nutrient delivery, which consists of sucking bursts and respiration intervals (i.e., pauses). Each burst usually contains 6–12 suck cycles and a within-burst frequency of 2 Hz, followed by pause periods to accommodate respiration.^39^ Importantly, research has shown that sensory stimulation—whether olfactory, visual, auditory, or vestibular—can influence sucking behavior in young infants (e.g.,^40^^,^^41^). Generally, NNS behavior is influenced by environmental stimuli, with both familiar and novel features playing a role in modulating attentional engagement.^42^ For example, newborns often increase their sucking behavior when exposed to familiar voices or languages (e.g.,^1^), whereas their sucking may decrease in response to novel or cognitively demanding stimuli (e.g.,^41^). Indeed, the NNS method is frequently employed to gauge newborns’ inherent preference toward two distinct stimuli or their ability to discriminate between them.^43^^,^^44^^,^^45^

### Aim and predictions of the study

The first aim of this study was to investigate the relative weight of socio-emotional and linguistic prosodic cues in capturing newborns’ attention within an ecologically valid nursery rhyme. More in depth, we tested whether newborns’ attention varied depending on the presence of socio-emotional and linguistic prosodic information. Newborns were exposed to three versions of the same nursery rhyme: one narrated by an actress in a naturalistic manner (i.e., narrated condition), a second hummed by the same actress (i.e., hummed condition), and a third synthesized version of the rhyme (i.e., synthesized condition). The narrated version enabled us to explore newborns’ attention to both socio-emotional and linguistic prosodic cues. The synthesized version allowed us to investigate how emphasizing linguistic information while minimizing socio-emotional cues affects voice processing. Lastly, the hummed version of the nursery rhyme enabled us to explore the impact of socio-emotional prosodic cues in the absence of linguistic content. Although direct evidence of mothers using humming in interactions with their babies is scarce,^46^^,^^47^ humming has been proposed as a pre linguistic form of vocal communication, potentially serving early caregiving roles, such as soothing or bonding, in both individual and group contexts.^48^ Given the paucity of literature on the topic, we considered two competing hypotheses regarding the role of socio-emotional prosodic and linguistic cues in capturing newborns’ attention. If socio-emotional prosody is critical for orienting attention, its absence in the synthesized condition may render the stimulus more perceptually novel, potentially eliciting stronger attentional responses—such as sucking suppression due to increased cognitive load or an orienting response. On the other hand, if linguistic content is more effective in capturing newborns’ attention, we would expect to observe stronger attentional responses in the hummed condition, as it lacks linguistic content despite retaining prosodic cues. Between them stands the narrated condition, serving as a middle ground for our interpretations. While previous research has addressed related questions through highly controlled stimuli, we chose to focus on ecologically valid stimuli in order to avoid artificiality and to mimic the environment surrounding newborns.

As a second aim, we investigated the role of prenatal exposure to music, as well as talking and singing, in influencing newborns’ perceptual sensitivity to and processing of prosodic information.^49^^,^^50^ Examining individual differences in these prenatal auditory experiences would provide valuable insights into how prenatal acoustic exposure shapes early attentional mechanisms. For instance, greater prenatal exposure to maternal speech or singing may enhance newborns’ familiarity with prosodic cues, potentially reducing their attention to such cues postnatally, as they would be perceived as less novel. Supporting this idea, studies have shown that daily musical exposure during the final trimester of pregnancy^51^ and exposure to maternal speech^49^^,^^52^^,^^53^ can enhance infants’ speech-processing abilities. To address this hypothesis, we investigated daily musical and speech exposure during pregnancy through self-report retrospective questionnaires, appropriately developed to examine the frequency with which mothers had been talking during the day (e.g., for work, with friends, etc.), the frequency with which they talked directly to the womb, and whether they used to sing or listen to music through loudspeakers during the last 3 months before delivery.

Lastly, we investigated the relationship between NNS variables at baseline (i.e., the initial silent period with no auditory stimulation) to better understand the underlying factors influencing the mechanisms driving NNS behavior. Indeed, the majority of evidence in literature adopted NNS as a means of accessing infants’ attentional skills and explored how this spontaneous behavior changes with external stimulation^2^^,^^40^^,^^41^^,^^43^^,^^45^^,^^54^ or with age.^55^ Also, studies have linked NNS patterns with neurodevelopmental outcomes to identify children who may be at risk for developmental delays and offer early intervention.^56^^,^^57^ NNS behavior can be described through various temporal and pressure-related features associated with cycles, bursts, and amplitude. A cycle is defined as a single sucking peak within the observation window, while a burst comprises one or more consecutive cycles separated by less than 1.2 seconds. Amplitude refers to the average pressure of these sucking peaks, measured in cmH_2_O. Based on these definitions, several quantitative measures can be derived, including the number of cycles and bursts per trial, the number of cycles within each burst, burst duration, burst intervals, cycle intervals, and cycles per burst intervals. Together, these variables provide a detailed view of the rhythm and intensity of sucking activity and have been linked to attentional processes.^39^^,^^58^ Although previous studies have explored correlations between NNS variables,^56^^,^^58^ the cognitive correlates underlying these variables are still virtually unknown. This gap in the literature highlights a strength of our study: the exploration of the underlying mechanisms of NNS behavior. Characterizing these relationships is essential for deepening our understanding of NNS mechanisms, refining data interpretation, and enhancing its utility in assessing attentional processes and neurodevelopmental outcomes.

## Results

### NNS across baseline, narrated, hummed, and synthesized conditions

Newborns were presented with a silent period with no stimulation (i.e., baseline condition) followed by three stimulation conditions in which they were presented with a nursery rhyme narrated by an actress (i.e., narrated condition), hummed by the same actress (i.e., hummed condition), or narrated by a synthesizer (i.e., synthesized condition). Each condition (i.e., narrated, hummed, and synthesized) was repeated a minimum of 1 and a maximum of 4 times, counterbalanced across participants (see Methods section for further details). To investigate whether newborns’ sucking behavior differed in the three conditions compared to the baseline condition, we ran linear mixed models (LMMs) on each dependent variable (NNS cycles/trial, NNS bursts/trial, NNS cycles/burst, NNS burst duration, NNS burst interval, NNS cycle interval, NNS cycles/burst interval, and NNS amplitude). In each model, predictors were conditions (baseline, narrated, hummed, and synthesized) and the random intercept was set to represent individual differences in sucking behaviors across subjects. Effect sizes were calculated with Cohen’s D. For each linear mixed-effects model, we assessed the normality of the dependent variables using Shapiro-Wilk tests. Results indicated that for the NNS cycles/trial and NNS cycles/burst interval variables, data were normally distributed within the four conditions (all *ps* > 0.07). By contrast, for the NNS cycles/burst and NNS burst duration variables, data in the baseline condition showed deviations from normality (*ps* < 0.001), while the other three conditions were normally distributed. LMMs are reportedly robust to normality violations, particularly when random effects are included.^59^^,^^60^ Nevertheless, to further ensure the robustness of our conclusions, we ran non-parametric tests, i.e., Friedman and Aligned Rank Transform (ART) analysis, an approach previously used with newborns data.^61^

**Figure 1:**
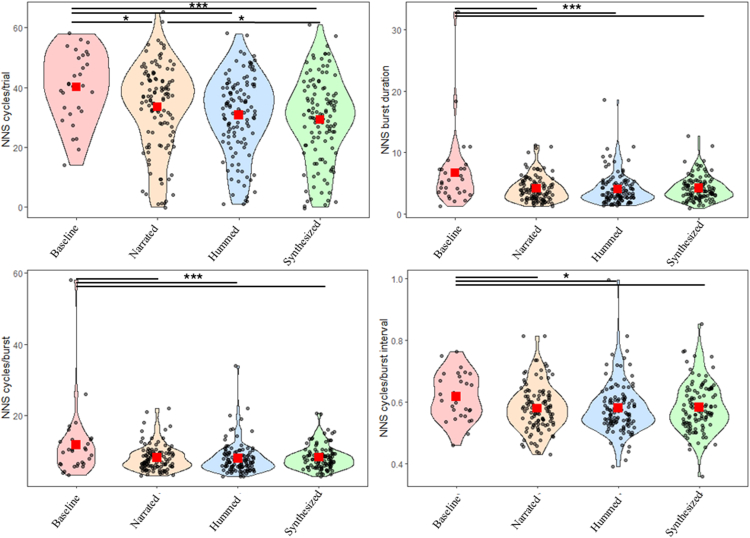
NNS across baseline, and narrated, hummed, and synthesized conditions Single data points of each subject for NNS cycles/trial (top left), NNS burst duration (top right), NNS cycles/burst (bottom left), and NNS cycles/burst interval (bottom right) for baseline, narrated, hummed, and synthesized conditions. The red square represents the group mean. ∗*p* < 0.05. ∗∗*p* < 0.005. ∗∗∗*p* < 0.001.

For the parametric analyses, we found a main effect of condition only for the model of NNS cycles/trial, F (3, 350.9) = 9.40, *p* < 0.001, with newborns sucking less for the synthesized (M = 29.1, SD = 12.6, t = 4.95, *p* < 0.0001, effect size = 0.8), the hummed (M = 30.6, SD = 12.6, t = 4.26, *p* = 0.0002, effect size = 0.69), and the narrated (M = 33.2, SD = 12.6, t = 3.09, *p* = 0.01, effect size = 0.5) conditions than the baseline (M = 40.2, SD = 30.1), and sucking less for the synthesized compared to the narrated condition (t = 2.92, *p* = 0.02). A main effect of condition was found for the NNS cycles/burst model, F (3, 329.4) = 7.83, *p* < 0.001, with fewer cycles in burst for synthesized (M = 8.26, SD = 4.48, t = 4.29, *p* = 0.0001, effect size = 0.77), the hummed (M = 7.98, SD = 4.34, t = 4.66, *p* < 0.0001, effect size = 0.83), and the narrated (M = 8.16, SD = 4.44, t = 4.21, *p* = 0.0001, effect size = 0.79), conditions than the baseline (M = 11.79, SD = 12.16). Also, we found a main effect of condition for the model of NNS burst duration**,** F (3, 329.4) = 11.07, *p* < 0.001, with shorter burst duration for synthesized (M = 4.25, SD = 2.65, t = 5.11, *p* < 0.0001, effect size = 0.92), the hummed (M = 4.07, SD = 2.57, t = 5.51, *p* < 0.0001, effect size = 0.98), and the narrated (M = 4.17, SD = 2.62, t = 5.29, *p* < 0.0001, effect size = 0.95), conditions than the baseline (M = 6.74, SD = 7.21). Lastly, we found a main effect of condition for the NNS cycles/burst interval model, F (3, 329.4) = 3.36, *p* = 0.02, with shorter inter-cycle distance inside the burst for synthesized (M = 0.59, SD = 0.07, t = 2.65, *p* = 0.04, effect size = 0.38), the hummed (M = 0.58, SD = 0.07, t = 3.06, *p* = 0.01, effect size = 0.44), and the narrated (M = 0.58, SD = 0.08, t = 2.94, *p* = 0.01, effect size = 0.42), conditions than the baseline (M = 0.62, SD = 0.13). Figure 1 depicts the average values of significant NNS.

The non-parametric analyses yielded convergent results with the parametric ones.

Specifically, as the variables NNS cycles/burst and NNS burst duration showed deviations from normality in the baseline condition (i.e., NNS cycles/burst baseline W = 0.61, *p* < 0.001; NNS burst duration baseline W = 0.65, *p* < 0.001), we reran the linear mixed models using non parametric tests. We used the classical non-parametric Friedman tests, followed by pairwise post hoc Durbin-Conover comparisons in Jamovi software v.2.6.26 (https://www.jamovi.org). We also conducted an (ART) analysis using the ARTool package in R. This method is well suited for repeated measures designs, accommodates multiple factors and their interactions, and has been successfully applied in developmental samples (e.g.,^61^). Both analyses confirmed the same pattern of results as the original linear mixed-effects models, thus supporting the robustness of our conclusions despite minor violations of the normality assumption.

Specifically, the Friedman analysis revealed a main effect of condition for the model of NNS cycles/burst, χ2(3) = 9.58, *p* = 0.02, with fewer cycles in burst for synthesized (d = 2.03, *p* = 0.045), the hummed (d = 2.45, *p* = 0.016), and the narrated (d = 3.02, *p* = 0.003) conditions than the baseline, and for the model of NNS burst duration, χ2(3) = 14.8, *p* = 0.002, with shorter burst duration for synthesized (d = 2.79, *p* = 0.006), the hummed (d = 3.55, *p* < 0.001), and the narrated (d = 3.55, *p* < 0.001) conditions than the baseline.

In line, the two ART models on NNS cycles/burst and on NNS burst duration - with conditions as fixed effects, and a random intercept for participants—revealed a main effect of condition for the model of NNS cycles/burst, F (3, 328.5) = 4.99, *p* = 0.002, with fewer cycles in burst for the synthesized (t = 2.73, *p* = 0.03), the hummed (t = 3.84, *p* = 0.0009), and the narrated (t = 3.01, *p* = 0.01) conditions than the baseline, for the model of NNS burst duration, F (3, 328.5) = 6.81, *p* = 0.0002, with shorter burst duration for the synthesized (t = 3.28, *p* = 0.006), the hummed (t = 4.48, *p* = 0.0001), and the narrated (t = 3.68, *p* = 0.002) conditions than the baseline.

### Correlation results between prenatal factors and NNS variables

Another aim of the present study was to explore whether prenatal variables, such as the maternal vocal environment (measured by means of self-administered questionnaires, for further details see the Method section), impact newborns’ attention to socio-emotional and linguistic prosodic cues. Correlation analyses to answer this question focused on the prenatal and the NNS variables resulted to be significantly modulated by our conditions (i.e., NNS cycles/trial, NNS cycles/burst, NNS burst duration, and NNS cycles/burst interval). To explore whether prenatal factors could impact newborns’ sucking behavior across conditions (i.e., baseline, narrated, hummed, and synthesized conditions), we run Pearson correlations between NNS cycles/trial, NNS cycles/burst, NNS burst duration, and NNS cycles/burst interval and between the singing behavior during pregnancy, the practice of a musical instrument during pregnancy and the amount of vocal experience the mother produced either in general or directed to the womb per day, and produced in general per week (see Table 1).

**Table 1:** Summary of the parental questionnaire administered to the mothers

Question	Response options
How much time did you typically spend speaking during the day (e.g., for work/with friends etc.)?	- Less than 2 h - 2 to 4 h - 4 to 6 h - More than 6 h
Do you speak with your unborn child and how often did you speak to your unborn child during the week?	- Never - Every day - Almost every day - Sometimes
How many times per day did you typically speak to your unborn child?	- Once - Two or three times - More than three times
Does anyone in the household usually play a musical instrument (e.g., you and/or your partner)?	Yes/No
Does anyone in the household usually sing (e.g., you and/or your partner)?	Yes/No

The questionnaire aimed to assess prenatal exposure to speech and music during the last trimester of pregnancy.

**Figure 2:**
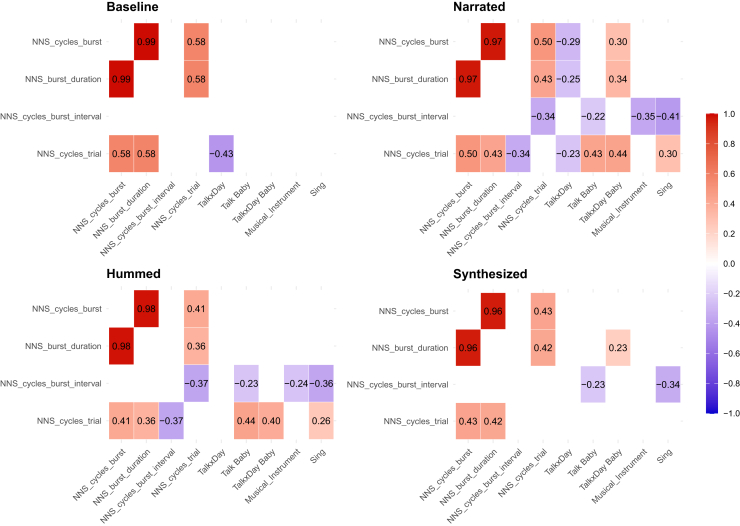
Correlations between prenatal factors and NNS variables Representation of the significant correlations in baseline (top left), narrated (top right), hummed (bottom left), and synthesized (bottom right) conditions between NNS variables and prenatal factors. Only significant correlations are shown in the figure (empty squares represent non-significant correlations). Correlations displayed in red indicate positive correlations, while those in blue represent negative correlations.

As illustrated in the correlation summary table (Figure 2), significant associations emerged across all conditions.

Specifically, in the baseline condition, we found that greater maternal vocal exposure reduces sucking behavior, as indicated by a negative correlation with the number of cycles per trial (r = −0.43, *p* = 0.04). On the contrary, in stimulation conditions maternal speech exposure was associated with increased sucking behavior. More in depth, in the narrated condition, talking to the womb was positively correlated with the number of cycles per trial (r = 0.44, *p* < 0.0002), the average number of cycles in each burst (r = 0.30, *p* = 0.005), and the length of the bursts (r = 0.34, *p* = 0.002), and negatively correlated with the inter-cycle distance inside the burst (r = −0.22, *p* = 0.045). Also, the amount of vocal experience not directed to the womb was negatively associated with the number of cycles per trial (r = −0.23, *p* = 0.03), the average number of cycles in each burst (r = −0.29, *p* = 0.007), and the length of the bursts (r = −0.25, *p* = 0.02). In the hummed condition, maternal talking to the womb was negatively associated with inter-cycle distance inside the burst (r = −0.23, *p* = 0.03) and positively associated with the number of cycles per trial (r = 0.44, *p* < 0.002). In the synthesized condition, maternal talking to the womb negatively correlated with the inter-cycle distance inside the burst (r = −0.23, *p* = 0.04) and positively with the length of the bursts (r = 0.23, *p* = 0.04).

We also found consistent effects of maternal singing and musical instruments in reducing newborns sucking behavior. In the narrated condition, singing and playing an instrument were negatively associated with the burst interval (e.g., singing: r = −0.41, *p* < 0.005; instrument: r = −0.35, *p* = 0.0006), while singing also showed a positive correlation with the number of cycles per trial (r = 0.30, *p* = 0.003). In the hummed condition, singing and playing an instrument were again negatively associated with burst interval (singing: r = −0.36, *p* = 0.0003; instrument: r = −0.24, *p* = 0.02), while singing showed a weak positive correlation with number of cycles per trial (r = 0.26, *p* = 0.01). Finally, in the synthesized condition, singing was negatively associated with inter-cycle distance inside the burst (r = −0.34, *p* = 0.001).

### PCA exploratory analysis on sucking variables: Characterizing NNS relationships in the baseline condition

**Figure 3:**
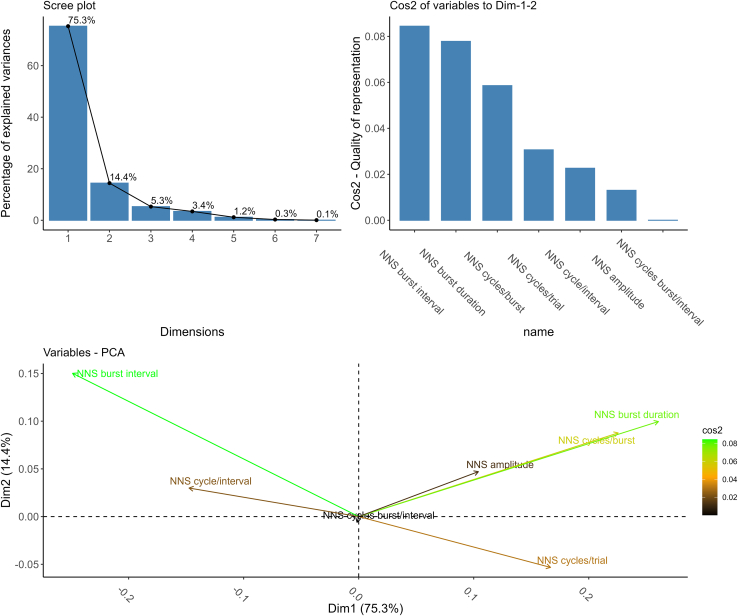
PCA on sucking variables (A) represents the scree plot, showing PCA eigenvalues, used to determine the number of components selected in the PCA. (B) shows the square cosine plot representing the correlation of the variables with each principal component. The same, with relative weights, is depicted through a biplot in (C).

To uncover patterns of relationships in NNS behavior, we performed an exploratory analysis across variables at baseline. To achieve this, we applied a principal-component analysis (PCA) to reduce data dimensionality and identify components that clarify the relationships among the variables. Our results highlight the presence of two components explaining 89% of variance in the data. The appropriate number of components was selected by visual inspection of the scree plot (Figure 3A). Upon examining the PCA component loadings (see Table 2), we interpreted these components as sucking effort—behavioral activation (Component 1) and inhibitory control—temporal regulation (Component 2). Overall loadings ranged from very poor to good across variables. This pattern likely reflects the fact that NNS behavior is influenced by multiple overlapping mechanisms (e.g., attentional mechanisms, inhibitory control, temporal synchronization, etc.), which are differentially captured by the two PCA components. Indeed, while some variables are more strongly associated with specific components (e.g., NNS cycles/trial with sucking effort and behavioral activation), others may have a weaker or more distributed relationship across components (e.g., NNS amplitude contributing in both components), possibly due to their role as intermediary or less dominant features of the underlying processes (see Discussion). The relative weight of individual variables across the two components is depicted in square cosine plot (Figure 3B) and in the biplot (Figure 3C).

**Table 2:** Component loadings across variable**s**

	Component 1 loading	Component 2 loading
	Sucking effort —behavioral activation	Inhibitory control—temporal regulation
**NNS Variables**		

*NNS_cycles_burst*	0.45	0.4
*NNS_burst_interval*	−0.5	0.69
*NNS_burst_duration*	0.53	0.46
*NNS_cycles_burst_interval*	−0.002	−0.02
*NNS_amplitude*	0.21	0.21
*NNS_cycle_interval*	−0.3	0.13
*NNS_cycles_trial*	0.33	−0.24

Components loadings of the PCA across non-nutritive sucking variables.

## Discussion

In the present study, we investigated newborns’ attention to socio-emotional and linguistic prosodic cues by presenting them with three versions of a nursery rhyme: one narrated by an actress in a naturalistic manner, incorporating both socio-emotional and linguistic prosodic cues (narrated condition), a second version hummed by the same actress, isolating and highlighting emotional prosodic cues (hummed condition), and a third synthesized version, preserving linguistic content but devoid of socio-emotional prosodic features (synthesized condition). Newborns’ attention was assessed through the NNS paradigm, which leverages the simple and rhythmic motor reflex of sucking—a fundamental ability found in all healthy infants.^38^

Overall, we found that newborns exhibited less sucking in the stimulation conditions (i.e., narrated, synthesized, and hummed) compared to the initial silent period (baseline). Specifically, there was a reduction in the number of peaks (NNS cycles/trials), the average number of cycles per burst (NNS cycles/burst), burst durations (NNS burst duration), and the interval between cycles within bursts (NNS cycles/burst interval) in the three stimulation conditions relative to the baseline, during which no auditory stimulation was provided. These findings replicate previous studies showing that the presence of salient stimuli captures newborns’ attention and compete for cognitive resources, leading to a reduction in both the frequency and duration of sucking behavior.^2^^,^^41^^,^^62^ However, other studies suggest that attentional engagement may enhance sucking behavior in newborns (e.g.,^1^^,^^47^). This apparent inconsistency in the literature reflects a broader theoretical debate regarding the functional meaning of sucking modulation in response to sensory stimulation.^41^^,^^44^^,^^45^ One interpretation posits that when attention is strongly engaged by novel or meaningful stimuli, motor systems may be transiently inhibited as part of a general “freezing” or orienting response, a mechanism that facilitates sensory processing and environmental monitoring by reducing self-generated movement.^41^^,^^62^ Conversely, other accounts propose that increases in sucking may signal attentional engagement, particularly in paradigms where sucking serves an active function—such as contingent sucking tasks, in which infants learn that increased sucking elicits preferred auditory stimuli.^1^^,^^2^ In these contexts, enhanced sucking reflects both motivation and goal-directed attention. Additionally, increases in NNS in response to emotionally salient or familiar stimuli may indicate arousal-driven attentional mobilization, rather than behavioral inhibition. Our results contribute to this debate by showing that, in a passive listening context, prosodic cues engage newborns and this engagement—interpreted as attentional—results in reduced sucking behavior. The origin of this inhibition phenomenon and its relationship to specific auditory properties of the stimuli (e.g., frequency, rhythm) is still debated. Zimmerman and Foran^41^ proposed that connectivity patterns between the auditory system and motor areas contribute to regulating sucking behavior. This network may synchronize with patterned orocutaneous stimulation, coordinating sucking behavior with external auditory stimuli, creating a form of sensory-motor synchronization.^63^ Although sensory-motor entrainment cannot be entirely ruled out, it is a less plausible explanation for our findings, as the stimuli presented in our study were too brief for such an effect to occur. Our results are, thus, better explained by referring to arousal mechanisms. As in previous studies linking neonatal behaviors—such as sucking and looking-time responses—to environmental monitoring,^41^^,^^45^^,^^62^ here the significant inhibition observed during all nursery rhyme presentations relative to baseline strongly indicates that salient environmental changes trigger motor inhibition, reinforcing the notion that environmental changes markedly influence motor responses in newborns.

Besides the presence of generalized salient changes in the environment, inhibition in our results is also modulated by the specific features of the auditory stimulation. Indeed our findings suggest that socio-emotional prosodic cues play a role in capturing newborns’ attention, as indicated by the inhibition of sucking behavior in the condition lacking these cues (i.e., the synthesized condition). Indeed, the lower number of NNS cycles in the synthesized condition compared to the narrated condition suggests that the artificial voice might be perceived as more complex, salient, or novel, potentially increasing cognitive load. In contrast, the narrated and hummed conditions preserve socio-emotional cues and may be perceived as more familiar and typical. More in depth, this suggests that beyond the general effect of introducing an auditory stimulus, the salience and characteristics of the stimulus itself play a critical role in shaping sucking behavior, with more salient stimuli eliciting a stronger inhibitory response. This suggests that the artificial voice may be perceived as less natural than the human voice, an effect also reported in adults.^64^^,^^65^^,^^66^ Thus, the inherent saliency and novelty of the synthesized voice could require increased cognitive load for its processing, especially for newborns. Strengthening this hypothesis, we did not observe a significant difference between the humming and narrated conditions, suggesting a pivotal role in socio-emotional cues, preserved in the humming condition, in modulating NNS behavior. Overall, these findings emphasize the critical role of socio-emotional cues in shaping early auditory experiences, guiding newborns’ attentional engagement and their interactions with the environment. Future research could further explore how different prosodic features interact to shape newborns’ attentional responses, helping to disentangle the relative contributions of each factor in perceptual and cognitive development.

Regarding the role of prenatal auditory exposure and its relationship with prosodic cues, we observed that the prenatal auditory environment—including exposure to maternal speech, music, or singing—may uniquely shape perceptual sensitivity and influence differences in newborns’ attentional skills. An exploratory aim of the present study was to investigate how these individual variations in prenatal auditory experiences, as reported by mothers, relate to newborns’ attentional responses across different auditory conditions (baseline, narrated, hummed, and synthesized). Our findings suggest that the extent and nature of prenatal exposure to musical instruments, singing, and speech may differentially impact how newborns engage with and process speech stimuli. Specifically, we found that in the baseline condition, in the absence of any stimulation, maternal speech shapes newborns’ attentional allocation, with more speech exposure reducing sucking behavior. This resonates with data showing that newborns’ brains are tuned to speech at birth and are more active during speech compared to silence.^3^^,^^6^ Also, we found that greater maternal speech exposure was associated with increased sucking behavior across experimental conditions. In this sense, greater prenatal exposure could familiarize newborns with speech-related auditory patterns, resulting in decreased attentional resources allocated to their processing. Interestingly, these results suggest that prenatal exposure to mother’s speech may establish a foundation for efficiently processing subsequent speech related stimuli and promoting auditory processing.^67^^,^^68^ In addition, we also observed a similar pattern for singing and musical instruments. This is consistent with evidence showing that music can scaffold language learning by enhancing the encoding of rhythmic and prosodic elements of the speech signal.^69^ Overall, although exploratory, these results suggest that newborns with greater prenatal exposure to speech and music may process auditory stimuli more efficiently, demonstrating reduced motor inhibition and increased sucking behavior.

Finally, given the complexity of NNS, which is shaped by multiple overlapping factors such as arousal, motor inhibition, and temporal regulation, we performed an additional exploratory analysis using PCA to uncover patterns of relationships among variables at baseline and to identify key components that could better clarify the relationships within the observed sucking patterns. Although exploratory in nature, the PCA revealed that two latent dimensions may underlie the structure of NNS behavior. However, these findings should be interpreted with caution as the second component accounted for a limited portion of variance (14%) and many factor loadings were relatively similar across variables, suggesting that the components are not fully distinct. We identified two components underlying NNS behavior: sucking effort—behavioral activation and inhibitory control—temporal regulation. The first reflects motor intensity and persistence, driven by variables such as burst duration, cycles per burst, cycles per trial, and amplitude—markers of arousal and attentional engagement. The second captures the coordination between effort and pause timing (e.g., burst/interval, cycles/interval), suggesting self-regulation and inhibitory control. Shared contributions of effort and timing variables across components point to integrated sensory-motor mechanisms, consistent with prior research on neonatal alertness and rhythmic synchronization with environmental cues.^41^^,^^56^^,^^58^^,^^63^^,^^70^ Although these findings provide a first attempt to characterize the NNS behavior at birth and the underlying attentional mechanisms, the results should be regarded as preliminary and further validated in future studies. Notably, different variables revealed similar contributions across both components, indicating that they may not represent a defining feature of either latent dimension.

In conclusion, our study provides novel and compelling evidence that newborns’ sucking behavior is modulated both by prenatal auditory experience and by the content of the stimuli, with the absence of socio-emotional prosodic cues capturing neonates’ attention the most. This finding sheds light on the critical role of prenatal auditory exposure in shaping early attentional mechanisms, suggesting that motor inhibition during sucking behavior is closely tied to how attentional resources are allocated. Beyond contributing to a deeper understanding of newborns’ behavioral responses, these results have profound implications for early language acquisition, offering new insights into how prenatal experiences may lay the foundation for cognitive development and the processing of socio-emotional cues in infancy.

### Limitations of the study

Comprehensively, ours is one of the few studies revealing the crucial role of socio-emotional prosodic and linguistic cues in voice perception in the first hours of life. Nonetheless, this study has some limitations that could restrict interpretation of our findings. The first issue regards our sample size. While far from small, especially considering the population of interest, our sample size was planned based on our primary aims, to investigate newborns’ attention across speech conditions compared to the baseline. As such, the sample size limits the ability to conduct more sophisticated secondary analyses requiring larger sample sizes (e.g., k-means clustering instead of PCA) which may reveal more nuanced relationships across NNS variables. Sample size limitations also extend to our exploratory analyses examining correlations between sucking variables and prenatal variables, where correlations were generally moderate to low. As such, it is important to interpret our correlations with caution. Indeed, missing data in maternal questionnaire responses—which reduced the sample size in some analyses—is influenced by different factors, such as maternal fatigue. Another limitation pertains to the assessment of prenatal auditory experiences, which relied on self-report measures. Future studies might overcome this issue by either manipulating the prenatal environment—such as encouraging mothers to enrich it with socio-emotional prosodic stimulation—or by refining the measurement of prenatal auditory exposure through more ecologically valid methods, for example, employing audio recording devices to capture the richness and variability of the fetal sound environment. In addition, to increase control on stimuli characteristics, future research could include an additional control condition in the form of vocoded speech by applying targeted acoustic manipulations. Although such approaches may compromise ecological validity, they would allow for more precise isolation of the contribution of specific components that guide early attentional engagement. A final issue in the present study is the exclusive reliance on behavioral measures to explore infants’ attention and behavioral alertness. While these measures provide valuable insights, future studies could delve deeper into the neural underpinnings of infants’ socio-emotional auditory processing by employing brain imaging techniques. Nonetheless, beyond the necessarily present limitations, this study provides useful insights on newborns’ attention to process socio-emotional and linguistic prosodic cues.

## Resource availability

### Lead contact

Further information and requests for resources should be directed to and will be fulfilled by Valentina Silvestri (valentina.silvestri@unimib.it).

### Materials availability

This study did not generate new unique reagents.

### Data and code availability

• Raw data have been deposited at OSF and are publicly available as of the date of publication. The DOI is https://osf.io/ysvr7/?view_only=bfe706f534db4a39b66aec4a20f0bae9.
• All original code has been deposited at OSF and is publicly available as of the date of publication. DOIs: https://osf.io/ysvr7/?view_only=bfe706f534db4a39b66aec4a20f0bae9.
• Any additional information required to reanalyze the data reported in this paper is available from the lead contact upon request.

## Acknowledgments

We would like to thank all the families and newborns that took part in the studies. Moreover, we are grateful to Carlo Toneatto for helping with the experiment creation and Nicole Miller-Viacava for providing the code used to compute the Modulation Power Spectrum and for insightful guidance on its theoretical foundations. This work was supported by a grant from the 10.13039/100031478Italian Ministry of University and Research—NextGenerationEU (PNRR M4.C2.I1.1—Avviso 104/2022, CUP H53D23004110006, grant no 2022-NAZ- 0410) awarded to Hermann Bulf.

## Author contributions

Conceptualization, V.S., S.P., and H.B.; methodology, V.S., S.P., and M.S., and H.B; formal analysis, V.S. and S.P.; investigation, V.S.; visualization, V.S. and S.P.; writing – original draft, V.S., S.P., and H.B; writing – review and editing, V.S., S.P., H.B, M.L.G., A.P., M.S., and L.C.; resources, M.L.G., A.P., L.C., and H.B.; supervision, H.B.

## Declaration of interests

The authors declare no competing interests.

## Declaration of generative AI and AI-assisted technologies in the writing process

During the preparation of this work the author(s) used ChatGPT 4 in order to improve the writing style of our manuscript. The authors reject using AI for scientific content creation. However, the authors believe that it helps foster the equality of native and non-native English speakers in order for them to have the same opportunities. After using this tool/service, the authors reviewed and edited the content as needed and took full responsibility for the content of the publication.

## STAR★Methods

### Key resources table

**Table:** 

REAGENT or RESOURCE	SOURCE	IDENTIFIER
**Deposited data**		

Raw and analyzed data	This paper	https://osf.io/ysvr7/?view_only=bfe706f534db4a39b66aec4a20f0bae9

**Software and algorithms**		

Jamovi software v.2.6.26	The jamovi project (2025)	https://www.jamovi.org
Praat	Paul Boersma & David Weenink 1992–2025	https://www.fon.hum.uva.nl/praat/
Python	Python Software Foundation	https://www.python.org/
Modulation Power Spectrum (MPS)	Miller-Viacava and colleagues^71^	https://www.biorxiv.org/content/10.1101/2025.03.04.638661v1.abstract
MATLAB	Mathworks, Natick, MA; ver. 2022b	https://www.mathworks.com/products/matlab.html
R	The R project	https://www.r-project.org/

### Experimental model and study participant details

#### Participants

A total of 30 healthy, full-term newborns (14 females; mean age = 51.9 hours, range = 24.4-86.8 hours) completed the study. An additional 14 infants were recruited and tested but were later excluded due to drowsiness/fussiness (*N* = 11), technical program errors (*N* = 2), or refusal of the pacifier (*N* = 1). Sample size was predetermined to reach larger sample sizes compared to previous studies investigating newborns’ attention to auditory stimuli using a similar design used in the present study (i.e., *N* = 12, see^36^) to mitigate potential power issues. We did not perform a power analysis before running the study. Still, we conducted a sensitivity power analysis using the SIMR package^72^ to determine the smallest effect size detectable with our sample size. This is not an observed/retrospective power analysis,^73^ as we critically manipulated the fixed effect sizes to determine the smallest detectable effect with 80% power. The results showed that our sample size would have allowed us to detect a difference of 6.5 cycles per trial in the synthesized condition with 80% power.

Newborns were recruited from the Neonatology and NICU Unit at the Fondazione IRCCS Ca’ Granda Ospedale Maggiore Policlinico. They all met the screening criteria of gestational age >37 weeks, a birth weight above 2300 g, and an Apgar score of at least 9 at 5 min, and all were tested one hour before feeding when they were in an awake and alert state. They all came from middle-class families and their parents self-reported as White and all exclusively Italian-exposed *in utero*. None of the participants reported the daily use at home of any kind of voice assistant with synthesized voice (e.g., Amazon, Google home, etc.).

Parental written informed consent was obtained before testing began. The protocol was carried out in accordance with the ethical standards of the Declaration of Helsinki (BMJ 1991; 302: 1194), and approved by the Ethics Committee (Comitato Etico Milano Area 2; ID: 694; Approval N. 952_2021).

### Method details

#### Stimuli

**Figure 4:**
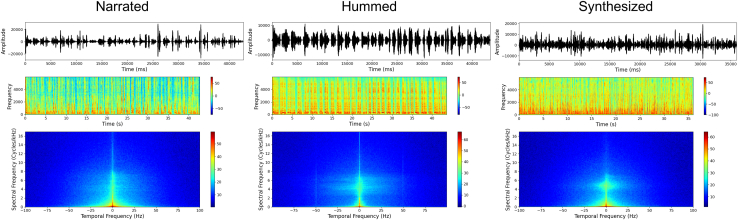
Graphical representation of the basic acoustic properties of stimuli (A–C) For each condition, the figure displays three key aspects of the signal: (A) the amplitude waveform, illustrating variations in sound intensity over time; (B) the frequency spectrum, representing the distribution of energy across different frequency components; and (C) the Modulation Power Spectrum (MPS), capturing the temporal and spectral modulation patterns that characterize each stimulus. Horizontal slices of the spectrogram (B) represent how the amplitude evolves over time at a specific frequency, while vertical slices at particular time points reveal spectral contrasts—that is, changes in the overall spectral power. However, speech structure is not made up of isolated spectrotemporal events; rather, it consists of sinusoidal-like patterns that extend across both time and frequency, often spanning multiple frequency bands and longer durations.^75^ These structured patterns encode crucial linguistic information and are represented in the MPS (C).^75^

Stimuli were represented by the nursery rhyme ‘Il maestro giusto’ by the Italian writer Gianni Rodari, presented in three forms: narrated by an actress, played on a synthesizer, and hummed by the same actress. The actress was an Italian native speaker, who provided both spoken and hummed stimuli. Specifically, for the narrated version, the actress was instructed to narrate the nursery rhyme in a natural tone of voice, simulating a soft and affectionate style commonly used when speaking to infants. The aim was to convey socio-emotional prosody while maintaining ecological validity. Then, the actress was instructed to hum the nursery rhyme, producing the text with a closed mouth while maintaining the main emotional prosodic features. Humming is usually adopted to generate melodies with sustained nasal sounds that have low spectral amplitude,^48^ useful to preserve the melodic components while disrupting linguistic information. To obtain a stimulus with preserved linguistic content but devoid of emotional prosodic features, we fed the text of the nursery rhyme into a phoneme-based text-to-speech synthesizer using formant synthesis. As a result, the stimulus had a flat, machine-like tone, devoid of emotional richness. These manipulations led to three audio recordings all 43 seconds in duration. Stimuli were preprocessed in Audacity 3.0.3, where noise and clicks in the audio recording were reduced. Then, using Praat (v6.1.37), stimuli were matched to the narrated condition intensity (66.13 dB) and pitch range (76.0 - 457.12 Hz) to make them comparable regarding low-level characteristics. As speech prosody is conveyed through several acoustical speech features, attempting to strictly control differences between stimuli—such as precisely matching pitch contours—would have altered their emotional content and resulted in stimuli that sound unnatural and lack ecological validity.^74^ Thus, we restricted the editing to these two operations to keep stimuli as naturalistic as possible and to preserve emotional cues from voices. To quantify the power of temporal and spectral modulations, and to ensure stimuli did not produce confounds due to their basic acoustic properties, we referred to the Modulation Power Spectrum (MPS)—computed as the squared magnitude of the two-dimensional Fourier transform of the log-magnitude spectrogram,^75^^,^^76^ following the code provided in Miller-Viacava and colleagues^71^ (Figure 4; see the caption for the details of the MPS calculation in Python, https://www.python.org/). Our resulting matrices were then averaged over either time or frequency rates and compared across conditions through t-tests. No significant differences were found in any comparison (lowest *p* = 0.41), indicating that the acoustic properties did not differ systematically across conditions and are unlikely to have introduced confounds in our data.

#### Apparatus and procedure

Approximately one hour before the planned feeding time, parents brought their infants to the laboratory inside the Neonatology Unit of the Hospital. Previous research has demonstrated that neonatal reflexes, such as sucking, are linked to the infant's capacity to achieve and sustain an alert state, hence this period was specifically selected to ensure that babies would be in an attentive state and ready to participate in the study.^38^^,^^41^^,^^45^ Newborns sat on an experimenter’s lap in a semi-inclined position. By softly caressing the baby’s cheek, the experimenter would provide the feeding bottle with the personalized pacifier attached. Registration began as soon as the newborn put the pacifier in his or her mouth. Every subject used a pacifier just once, and it was thrown away after each use. The researcher holding the newborn was instructed not to wear any perfume or any personal care products and not to engage or look at the infant during the testing, trying to place the head backward so as not to be seen by the newborn during the entire session.

A second researcher was present in the room and in control of the data acquisition system, making sure that the data was appropriately obtained by the apparatus. One or both parents were present during the entire experimental session, seated behind the experimenter and out of the infant’s direct line of sight, and could interact with the baby only if they manifested discomfort, thus interrupting the experimental session. Lights in the room were dimmed for the duration of the experiment.

**Figure 5:**
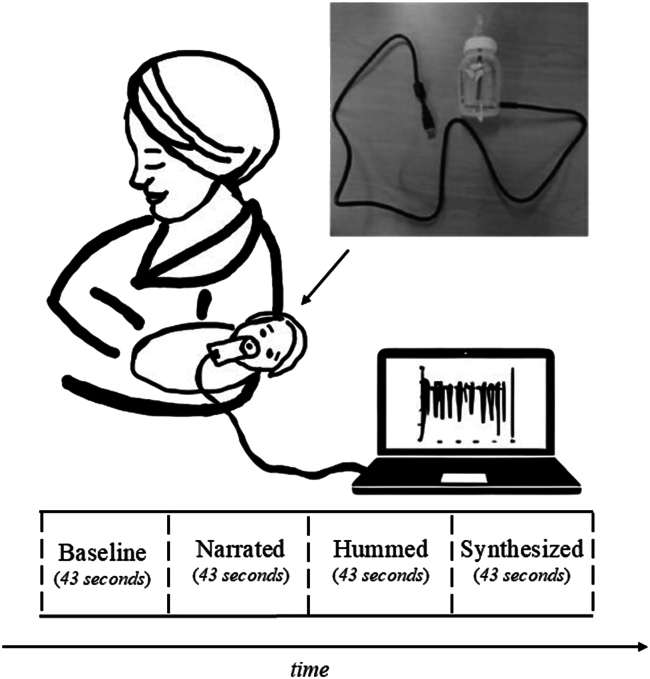
Schematic representation of the experimental session and setup, including the customized pacifier The figure illustrates the timeline of the experimental session. A photograph of the customized pacifier device used to measure non-nutritive sucking (NNS) is included.

Stimuli were presented free field at an intensity of 70 dB via speakers of the computer. Each experimental session began with an initial 43-second silent baseline period, followed by three stimulation conditions of equal duration: nursery rhymes narrated by the actress, hummed by the actress, and narrated by the synthesizer. Each of the three stimulation conditions (i.e., narrated, hummed, and synthesized) was randomly repeated to the newborn up to 4 times or until he/she showed signs of discomfort. A session was considered valid if the infant is presented, after baseline, with at least one repetition per condition. To prevent order effects, the sequence of the three stimulation conditions was pseudo-randomized and counterbalanced across participants (see Figure 5). Therefore, the experimental session could have a minimum duration of approximately three minutes/four trials (i.e., baseline – narrated– hummed – synthesized) and a maximum of nine and a half minutes/thirteen trials. On average, newborns completed 12.8 trials (SD = 1.10).

The Non-Nutritive Sucking behaviour was recorded via a feeding bottle holding a custom pacifier, inside which was placed a pressure transducer, connected to a computer. The raw signal was processed using MATLAB (Mathworks, Natick, MA; ver. 2022b), by subtracting the value of the resting pressure from the entire signal and down-sampled at 100 Hz. A band-pass filtered (0.5-20 Hz) in order to remove low-frequency off-sets due to tongue/jaw posturing, thermal drift associated with the contact between the oral apparatus and the pacifier bulb, and the high-frequency jitter.^70^^,^^77^ Lastly, a smoothing was performed as in Barlow and colleagues.^70^ Cycles and bursts identification was performed using the findpeak function in MATLAB. An event was considered a cycle if pressure exceeded 1.5 mbar.^70^^,^^77^^,^^78^ A NNS burst was defined as 2 or more suck cycles satisfying a cycle period of 1.2 ms.^70^ From NNS behavior, we extracted different variables: NNS cycles/trial (i.e., number of peaks/sucks in each time window), NNS bursts/trial (i.e., one or more peaks satisfying a cycle period less than 1.2 ms), NNS cycles/burst (i.e., average number of peaks in each burst), NNS burst duration (i.e., length of the bursts in seconds), NNS burst interval (i.e., distance between bursts), NNS cycle interval (i.e., distance between peaks), NNS cycles/burst interval (i.e., inter-cycle distance inside the burst) and NNS amplitude (i.e., average pressure of cycles in cmH20).

The musical and speech exposure that newborns underwent during pregnancy was assessed by a short retrospective questionnaire delivered to the babies’ mothers (see Table 1). Based on previous research,^79^^,^^80^^,^^81^^,^^82^ mothers were asked the frequency with which they talk during the day (e.g., for work, with friends, etc.), the frequency with which they talked directly to the womb, and whether they used to sing or listen to music through loudspeakers during the last 3 months before delivery. Specifically, they were asked to indicate whether they talked during the day less than 2 hours, from 3 to 4 hours, from 4 to 6 hours, or more than 6 hours. They were asked to indicate the average frequency of talking directly to the womb during the week (i.e., sometimes, almost every day, or every day) and during the day (i.e., one time per day two times a day, or more than 3 times a day). Furthermore, they were asked to indicate whether they (or the partner) were singing or playing an instrument every day (i.e., by answering either 'yes' or 'no'). Out of the 30 participants in the study, 23 mothers (76.7%) completed the questionnaire.

### Quantification and statistical analysis

To investigate whether the newborns sucking behavior differed in the three conditions (i.e., narrated, hummed, and synthesized) compared to the baseline condition, we ran linear mix models (LMM) analyses on each dependent variable (NNS cycles/trial, NNS bursts/trial, NNS cycles/burst, NNS burst duration, NNS burst interval, NNS cycle interval, NNS cycles/burst interval, and NNS amplitude) including the predictor condition (baseline, narrated, hummed, and synthesized) and a random intercept for each subject to account for individual differences in sucking behavior. We used the lme4 package^59^ in R (version 4.2.3), with default Satterthwaite approximations for degrees of freedom (usually suitable for datasets with smaller numbers of subjects and observations).

To explore whether prenatal variables impact newborns’ attention through sucking behavior, we correlated NNS variables resulted to be significantly modulated by our conditions (i.e., NNS cycles/trial, NNS cycles/burst, NNS burst duration, and NNS cycles/burst interval) and prenatal variables in each condition, that is in the baseline and/or in the narrated, hummed and synthesized conditions. For the correlations between continuous variables (e.g., NNS cycles/trial and Talk x Day), we used Pearson’s correlation, while for the binary variables (e.g., Sing and Musical Instrument), we used point-biserial correlation. Correlations were calculated using the function *corr.test()* implemented in the R-library *psych*.^83^ Given the number of comparisons, their significance level was adjusted using the False Discovery Rate.^84^

To uncover relationships across NNS variables and reduce data dimensionality we performed a PCA. First, the data were normalized to a common logarithmic scale. We then performed a visual inspection of the scree plot to evaluate the importance of each extracted principal component (Figure 3). After identifying the optimal two-component solution that explained the most variance in the data, we extracted these components and examined their loadings. All steps were performed in R (version 4.3.3) through the *factoextra* package^85^ and the *princomp()* function.
